# Impact of Androgen Deprivation Therapy on Lower Urinary Tract Symptoms in Patients with Prostate Cancer

**DOI:** 10.3390/medicina62020359

**Published:** 2026-02-11

**Authors:** Tae Young Park, Sung Ryul Shim, Chang Hee Kim

**Affiliations:** 1 Gil Medical Center, Department of Urology, College of Medicine, Gachon University, Incheon 21936, Republic of Korea; 1033163@gilhospital.com; 2 Department of Biomedical Informatics, College of Medicine, Konyang University, Daejeon 35365, Republic of Korea; sungryul.shim@gmail.com

**Keywords:** prostate cancer, androgen deprivation therapy, international prostate symptom score, prostate volume, quality of life, lower urinary tract symptoms

## Abstract

*Background and Objectives*: The increasing number of patients with prostate cancer receiving long-term androgen deprivation therapy (ADT) underscores the importance of maintaining quality of life during treatment. Lower urinary tract symptoms (LUTS), influenced by prostate size, represent significant determinants of quality of life in this population. This study aimed to investigate the impact of ADT on LUTS in patients with prostate cancer, particularly focusing on how ADT, which reduces prostate volume (PV), affects quality of life, and to identify factors influencing changes in LUTS. *Materials and Methods*: The study included 104 patients with prostate cancer undergoing ADT. Changes in the International Prostate Symptom Score (IPSS), PV, maximal uroflow rate (Qmax), post-void residual urine volume (RU), and prostate-specific antigen (PSA) levels were compared before treatment initiation and at 12 and 24 weeks after treatment. Association between these variables and patient age, body mass index (BMI), Gleason score (GS), and T stage were also assessed. *Results*: After 12 and 24 weeks of ADT, prostate size decreased by 16.69 cm^3^ (32.03%) and 25.36 cm^3^ (48.68%), respectively, with PSA levels decreasing by 7.63 ng/mL and 22.03 ng/mL. Qmax improved by 3.19 mL/s and 5.57 mL/s, and RU decreased by 42.31 mL and 60.68 mL, respectively (*p* < 0.001). The IPSS decreased by 13.09 and 14.69 points at 12 and 24 weeks, respectively (*p* < 0.001). Notably, patients with moderate-to-severe LUTS (baseline IPSS ≥ 8) showed a significantly greater reduction in IPSS (*p* < 0.001). Additionally, patient age and PSA levels were significantly associated with changes in Qmax (*p* < 0.001). *Conclusions*: ADT demonstrated a positive effect on LUTS improvement in patients with prostate cancer, particularly among those with moderate-to-severe LUTS, elevated PSA levels, or older age.

## 1. Introduction

Prostate cancer represents the most common and rapidly increasing cancer among men [1,2]. In recent years, the number of patients with prostate cancer undergoing androgen deprivation therapy (ADT) has significantly increased [1]. ADT is recommended for patients showing increased prostate-specific antigen (PSA) levels following prostatectomy or radiation therapy, as well as for individuals with asymptomatic metastasis, node-positive status, or radiological evidence of metastasis, and or advanced-stage prostate cancer [3].

Improved survival among patients with prostate cancer receiving ADT has resulted in a growing population undergoing long-term treatment [4]. Therefore, maintaining the quality of life (QoL) for patients with prostate cancer during ADT has emerged as an important issue [3]. However, ADT is associated with side effects such as libido loss, gynecomastia, erectile dysfunction, anemia, cardiovascular disease, osteoporosis, and depression, all of which can adversely affect the QoL [5].

Lower urinary tract symptoms (LUTS), frequently observed in older men, significantly affect the QoL. Previous studies have reported that 55.6% of patients with prostate cancer experience mild voiding problems, 37.1% experience moderate problems, and 7.3% experience severe problems [6]. Prostate volume (PV) serves as a major determinant of LUTS, and ADT is known to reduce both prostate cancer progression and prostate volume. Although changes in PV and LUTS are expected during ADT, few studies have comprehensively examined this relationship [7].

Therefore, this study aimed to investigate the effects of ADT on LUTS in patients with prostate cancer, identify the clinical factors contributing to changes in LUTS, and provide valuable insights into strategies for maintaining and improving QoL during ADT.

## 2. Materials and Methods

During the preparation of this work, the author(s) used AI-based tools (such as Gemini) solely to improve the language, grammar, and readability of the manuscript. After using these tools, the author(s) reviewed and edited the content as needed and take(s) full responsibility for the final version of the publication. These tools were not used to generate scientific data or to build core citation information.

### 2.1. Participant Recruitment

This exploratory study aimed to investigate changes in PV and LUTS in patients with prostate cancer undergoing ADT and to identify factors influencing these changes. The study was conducted at the Gil Medical Center and included 104 patients diagnosed with prostate cancer who were scheduled to receive ADT. The inclusion criteria were as follows: (1) patients without contraindications to ADT who were eligible to receive ADT for at least 6 months according to the treatment plan and had a life expectancy of at least 12 months; (2) patients who had not used urological medications, including alpha (α)-1 blockers or anticholinergic agents, or had discontinued them for at least 3 months before study initiation and did not use them during the study period; (3) patients capable of independently reading, understanding, and completing study questionnaires, with provision of written informed consent. The exclusion criteria were as follows: (1) patients who were undergoing or scheduled to undergo treatments other than ADT for prostate cancer (e.g., radiation therapy or prostatectomy); (2) patients with a history of prostate surgery for benign prostatic hyperplasia (BPH); (3) patients with an indwelling urinary catheter; (4) patients diagnosed with or treated for other malignancies.

All procedures adhered to the principles of the Declaration of Helsinki and were approved by the Gachon University Institutional Review Board (approval number: GCIRB 2020-066).

### 2.2. Measures of the Severity of Lower Urinary Tract Symptoms

The International Prostate Symptom Score (IPSS) is a widely utilized self-reported questionnaire for evaluating the severity of LUTS [8]. LUTS severity is categorized based on the total IPSS: mild (1–7), moderate (8–19), and severe (20–35) [9]. The IPSS voiding subscore (IPSS-V) is derived from the sum of responses to questions 1 (incomplete emptying), 3 (intermittency), 5 (weak stream), and 6 (straining). Similarly, the IPSS storage subscore (IPSS-S) is calculated from the sum of scores for questions 2 (frequency), 4 (urgency), and 7 (nocturia). Patient-reported QoL related to urinary symptoms is assessed by the eighth IPSS question, with scores ranging from 0 (delighted) to 6 (unhappy). All participants independently completed the IPSS questionnaire throughout the study period.

### 2.3. Study Design

The study initially recorded demographic and baseline clinical data, including age at diagnosis, body mass index (BMI), Gleason score (GS), and T stage, before the initiation of ADT. All patients received a luteinizing hormone-releasing hormone (LHRH) agonist (leuprolide depot 3.75 mg) subcutaneously every 4 weeks according to the established ADT schedule. Moreover, an anti-androgen agent (bicalutamide 50 mg) was administered for 1 month to prevent disease flare-up associated with initial leuprolide administration.

Before ADT initiation, each patient completed the IPSS questionnaire to obtain baseline total, voiding, and storage scores, along with QoL scores. Additionally, PV, maximal uroflow rate (Qmax), post-void residual urine volume (RU), and PSA levels were recorded. PV (mL) was measured using transrectal ultrasound (HS30, SAMSUNG MEDISON, Seoul, Republic of Korea) and calculated using the formula: Volume = width × length × height × 0.5236. Qmax was assessed using uroflowmetry (CUBEFlow_S, Mcube Technology, Seoul, Republic of Korea), and RU was evaluated via ultrasonography.

At 12 and 24 weeks after ADT initiation, IPSS (total, voiding, and storage), QoL, PV, Qmax, RU, and PSA levels were reassessed and compared with baseline values. Based on the baseline IPSS, patients were categorized into two groups: mild LUTS group (IPSS 1–7) and moderate-to-severe LUTS (IPSS ≥ 8). Changes in IPSS, PSA, PV, Qmax, and RU at 12 and 24 weeks after ADT initiation were compared between the two groups.

### 2.4. Statistical Analysis

The primary efficacy outcomes were changes from baseline to 12 and 24 weeks in PV, Qmax, RU, PSA, and IPSS. The primary analysis followed a per-protocol (completer) approach, excluding participants who withdrew or were lost to follow-up to assess treatment effects under full adherence. To address potential bias from missing data and to confirm temporal trends, we additionally implemented linear mixed-effects models (LMEM) with random intercepts for participants and fixed effects for time and key covariates. This model-based approach accounts for the inherent correlation between repeated measurements and effectively handles missing observations, ensuring the robustness of our longitudinal findings.

The normality of continuous variables was assessed using the Shapiro–Wilk test. Although variables largely satisfied normality assumptions, data are presented as medians with interquartile ranges (IQRs) to provide a robust description of the distribution, particularly considering the sample size disparity between the mild and moderate-to-severe LUTS groups.

Accordingly, non-parametric tests were employed for the primary inferential analysis. Comparisons between groups were performed using the Mann–Whitney U test, and within-group changes from baseline were analyzed using the Wilcoxon signed-rank test. Categorical variables were compared using the chi-square (χ^2^) test or Fisher’s exact test, as appropriate. To validate the statistical reliability against the sample size imbalance, sensitivity analyses using parametric tests (Student’s *t*-test and paired *t*-test) were also conducted, confirming the consistency of the significant results.

Predictors of clinically meaningful LUTS improvement were initially screened using univariate logistic regression; variables with *p* < 0.10 were entered into multivariable logistic regression models. Baseline PV was explicitly included as a key covariate to adjust for its potential influence on symptomatic and urodynamic improvements. Multicollinearity was evaluated using variance inflation factors (VIFs), and model fit was assessed via the Hosmer–Lemeshow test. When multiple comparisons were performed, *p*-values were adjusted using Bonferroni or Holm procedures. All tests were two-tailed, and statistical significance was set at *p* < 0.05. Statistical analyses were conducted using SPSS (version 29.0) and R (version 4.4.2).

## 3. Results

A total of 104 patients were included in the final analysis. The median age was 76.00 years (IQR, 71.00–80.25), and the median BMI was 23.34 kg/m^2^ (IQR, 21.78–26.08). Regarding Gleason score patterns, 14 patients (13.46%) had a score of ≤6 (3 + 3). For Gleason score 7, 5 patients (4.81%) presented with pattern 3 + 4, and 10 patients (9.62%) presented with pattern 4 + 3. Among patients with a score of ≥8 (n = 75, 72.12%), 30 (28.85%) had a score of 8 (4 + 4), 14 (13.46%) had a score of 9 (4 + 5), 18 (17.31%) had a score of 9 (5 + 4), and 13 (12.50%) had a score of 10 (5 + 5). The T-stage distribution included 27 patients (25.96%) with T2, 45 (43.27%) with T3, and 32 (30.77%) with T4 disease. Before ADT initiation, the median PV was 43.10 cm^3^ (IQR, 32.89–61.95), the median PSA level was 42.25 ng/mL (IQR, 16.42–100.00), the median Qmax was 9.10 mL/s (IQR, 5.10–15.25), and the median RU was 50.00 mL (IQR, 18.00–110.00). Furthermore, at baseline, the median IPSS total score was 23.00 (IQR, 13.00–29.00), the median IPSS voiding subscore was 14.00 (IQR, 6.00–17.00), the median IPSS storage subscore was 9.00 (IQR, 7.50–15.00), and the median QoL score was 4.00 (IQR, 4.00–5.00) (Table 1).

### 3.1. Changes in Variables: PV, PSA, Qmax, RU, and IPSS

**Figure 1:**
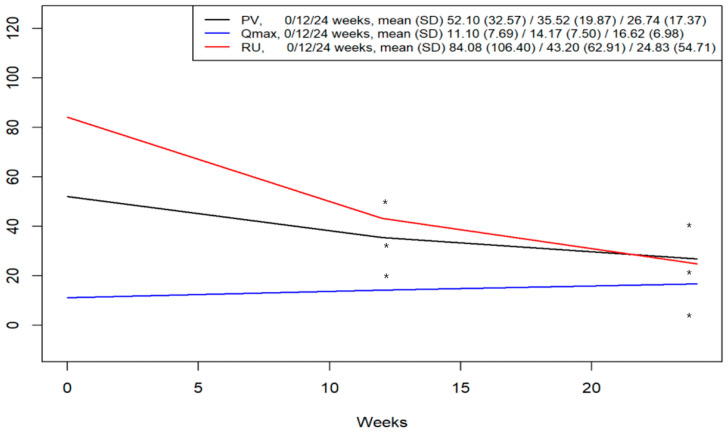
Median changes in prostate volume (PV), maximum urinary flow rate (Qmax), and residual urine volume (RU) with interquartile ranges (IQRs) from baseline. * *p*-value < 0.05 from baseline, Wilcoxon signed-rank test. IQR, interquartile range.

The median prostate volume (PV) at 12 and 24 weeks post-ADT initiation was 30.8 cm^3^ (IQR 22.1–41.6) and 23.0 cm^3^ (IQR 15.0–33.0), respectively. Compared to baseline (43.1 cm^3^, IQR 32.9–62.0), these values represented significant median reductions of 10.7 cm^3^ (IQR 1.5–24.0) at 12 weeks and 19.4 cm^3^ (IQR 8.6–35.9) at 24 weeks (*p* < 0.001 by Wilcoxon signed-rank test). The median PSA levels at 12 and 24 weeks post-ADT were 1.6 ng/mL (IQR 0.2–6.3) and 0.2 ng/mL (IQR 0.04–2.4), respectively, reflecting substantial median reductions from the baseline level of 42.3 ng/mL (IQR 16.4–100.0) (*p* < 0.001). Regarding Qmax, the median values at 12 and 24 weeks post-ADT were 12.2 mL/s (IQR 10.0–18.0) and 15.4 mL/s (IQR 12.7–18.6), respectively. These represented significant median increases from the baseline value of 9.1 mL/s (IQR 5.1–15.3) (*p* < 0.001). The median residual urine (RU) at 12 and 24 weeks post-ADT was 25.0 mL (IQR 0.0–50.0) and 0.0 mL (IQR 0.0–25.0), respectively, showing a significant decrease from the baseline median of 50.0 mL (IQR 18.0–110.0) (*p* < 0.001). Finally, the median IPSS total score significantly improved from 23.0 (IQR 13.0–29.0) at baseline to 8.0 (IQR 3.0–12.0) at 12 weeks and 7.0 (IQR 3.0–8.0) at 24 weeks (*p* < 0.001) (Figure 1).

**Figure 2:**
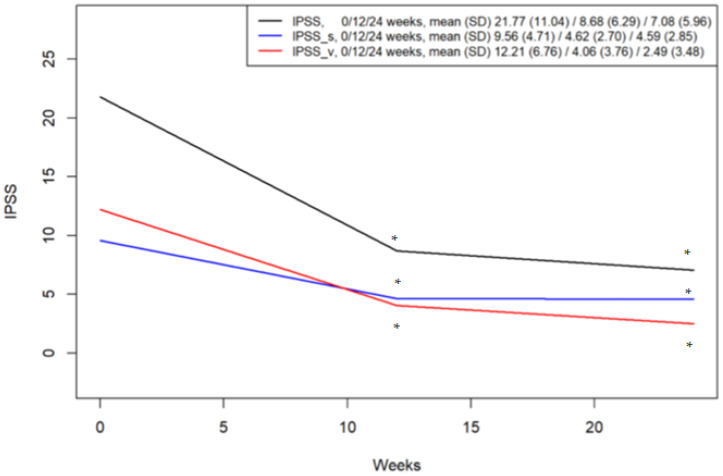
Mean changes in International Prostate Symptom Score (IPSS) with 95% confidence intervals from baseline. * *p*-value < 0.05 from baseline, paired *t*-test. SD, standard deviation.

The median IPSS total score at 12 and 24 weeks post-ADT initiation was 8.0 (IQR 3.0–12.0) and 7.0 (IQR 3.0–8.0), respectively. These values demonstrated significant median reductions from baseline of 14.0 points (IQR 6.8–22.2) at 12 weeks and 16.5 points (IQR 5.8–23.0) at 24 weeks (*p* < 0.001 by Wilcoxon signed-rank test). Further analysis of subscores revealed that the median IPSS voiding subscore decreased to 5.0 (IQR 0.0–6.0) at 12 weeks and 1.0 (IQR 0.0–3.5) at 24 weeks post-ADT, representing median reductions of 9.0 (IQR 4.0–14.0) and 11.0 (IQR 4.0–17.0) points from baseline, respectively (*p* < 0.001). Similarly, the median IPSS storage subscore also showed improvement, decreasing to 3.5 (IQR 2.0–6.0) at 12 weeks and 3.0 (IQR 3.0–6.0) at 24 weeks, corresponding to median reductions of 5.5 (IQR 2.0–9.0) and 6.0 (IQR 2.0–9.0) points, respectively (*p* < 0.001). Regarding patient-reported QoL, the median QoL score at 12 and 24 weeks post-ADT initiation was 2.0 (IQR 1.0–4.0) and 1.0 (IQR 1.0–2.0), respectively. These scores reflected median reductions from baseline of 1.0 point (IQR 0.0–3.0) at 12 weeks and 3.0 points (IQR 2.0–4.0) at 24 weeks (*p* < 0.001) (Figure 2).

### 3.2. Comparison of Changes Between Mild and Moderate-to-Severe LUTS Groups

Patients were categorized into a mild LUTS group (IPSS 1–7, n = 18) and a moderate-to-severe LUTS group (IPSS ≥ 8, n = 86) based on their baseline symptom severity. When comparing clinical outcomes between these cohorts, both groups demonstrated significant internal improvements from baseline in physiological and urodynamic parameters. Specifically, significant median decreases in PSA, PV, and RU, alongside significant median increases in Qmax, were observed in both groups at 12 and 24 weeks post-ADT (all *p* < 0.05 by Wilcoxon signed-rank test). However, the magnitude of these improvements (median changes from baseline) did not differ significantly between the two groups (all *p* > 0.05 by Mann–Whitney U test). Distinct and contrasting patterns were observed regarding patient-reported symptom scores (Table 2). In the moderate-to-severe LUTS group, the median IPSS total score showed a substantial decrease from 25.0 (IQR 22.0–35.0) at baseline to 7.0 (IQR 3.0–8.0) at 24 weeks (*p* < 0.001). Conversely, the mild LUTS group exhibited a significant increase in median IPSS total score from 3.0 (IQR 3.0–3.0) to 6.5 (IQR 3.0–8.5) during the same period (*p* < 0.001). Quality of life (QoL) scores improved significantly in both groups at 24 weeks post-ADT (*p* < 0.01). Notably, the median reduction in the QoL score was significantly more pronounced in the moderate-to-severe group (3.5 points, IQR 2.0–4.0) compared to the mild group (2.0 points, IQR 0.0–3.0) (*p* < 0.01 by Mann–Whitney U test), aligning with the greater symptom relief experienced by those with higher baseline scores.

### 3.3. Variables Affecting Lower Urinary Tract Symptoms

To identify the predictors of clinically meaningful LUTS improvement, univariate and multivariate logistic regression analyses were performed (Table 3). While several clinical factors were evaluated, including age, BMI, and baseline PSA, they did not reach statistical significance as independent predictors for IPSS improvement at 24 weeks in this cohort. However, multiple linear regression confirmed that baseline age and PSA values were significantly associated with the degree of Qmax change. Specifically, for every 1 ng/mL increase in baseline PSA, Qmax increased by 0.023 mL/s at 12 weeks (β = 0.0231, *p* < 0.001) and by 0.015 mL/s at 24 weeks post-ADT (β = 0.0154, *p* < 0.001). Additionally, for every 1-year increase in baseline patient age, Qmax significantly increased by 0.23 mL/s at 24 weeks post-ADT (β = 0.227, *p* = 0.043).

## 4. Discussion

This study investigated the impact of ADT on LUTS in patients with prostate cancer. After 12 and 24 weeks of ADT, patients demonstrated significant decreases in PV, PSA, and RU, whereas Qmax significantly increased. The mean IPSS total, IPSS voiding subscore, IPSS storage subscore, and QoL scores significantly decreased, indicating overall improvement. When patients were stratified into mild LUTS and moderate-to-severe LUTS groups based on their baseline IPSS, both cohorts exhibited significant reductions in PSA, PV, and RU, alongside an increase in Qmax at both 12 and 24 weeks post-ADT. However, improvements in IPSS total, IPSS voiding subscore, and IPSS storage subscore (i.e., significant decreases) were observed exclusively in the moderate-to-severe LUTS group. Regression analyses also revealed specific associations with changes in Qmax. For every 1 ng/mL increase in baseline PSA value, Qmax significantly increased by 0.023 mL/s at 12 weeks and by 0.015 mL/s at 24 weeks post-ADT. Additionally, for every 1-year increase in patient age at baseline, Qmax significantly increased by 0.23 mL/s at 24 weeks post-ADT.

The co-occurrence rate of LUTS in patients with prostate cancer remains considerably high [6]. Because prostate cancer commonly originates in the peripheral zone of the prostate, LUTS may not manifest in the early stages. As the cancer progresses and invades or compresses adjacent structures, such as the prostatic urethra or urinary bladder, LUTS often develop [10,11]. BPH, a well-established cause of LUTS, becomes increasingly prevalent with age, reflecting the parallel rise in prostate cancer prevalence among older populations [12,13]. Consequently, the proportion of patients with prostate cancer and coexisting BPH is typically high. Approximately 44.4% of patients with prostate cancer experience moderate-to-severe voiding problems attributed to BPH [7].

Several studies have reported that ADT significantly reduces PV in patients with prostate cancer [7,14,15,16,17]. For example, one study demonstrated that PV in patients with prostate cancer receiving ADT for 12 weeks decreased by an average of 37.5% [18]. Another study reported an average PV reduction of 51.14% when ADT was administered for less than 1 year [16,19]. These findings indicate that PV typically decreases by 30% to 50% on average following ADT. ADT may be administered as monotherapy with LHRH agonists or in combination with antiandrogens. Although LHRH agonist monotherapy resulted in a 32% reduction in PV, combination therapy involving antiandrogens achieved a 41.2% reduction, suggesting the latter is more effective in decreasing PV [16]. In this study, significant PV reductions of 32.03% at 12 weeks and 48.68% at 24 weeks post-initiation of combination ADT (LHRH agonists and antiandrogens) were confirmed in patients with prostate cancer compared to baseline. Importantly, the extent of PV reduction following ADT is closely related to the pre-ADT PV; larger baseline volumes tend to exhibit a more pronounced reduction effect [19]. This PV reduction effect of ADT surpasses that of 5-alpha reductase inhibitors (5ARIs), which are commonly used for BPH treatment and typically achieve an average PV reduction of 25% [18]. Therefore, besides its established role in reducing tumor burden in prostate cancer management, ADT appears to contribute meaningfully to the alleviation of LUTS through PV reduction [20].

LUTS demonstrate a negative association with Qmax and a positive association with RU. Numerous clinical studies have consistently shown that increased LUTS severity is associated with decreased Qmax and increased RU, indicating worsened bladder outlet obstruction and incomplete bladder emptying [21,22]. Furthermore, increased PV associates with reduced Qmax and elevated RU; therefore, exacerbating LUTS severity. Consequently, prostate size reduction is expected to improve LUTS by enhancing Qmax and decreasing RU [23]. Prior research has indicated that ADT effectively improves LUTS. For instance, patients with prostate cancer receiving ADT exhibited a 38% increase in Qmax and a 36% reduction in RU after 12 months [24]. Moreover, approximately 62% of patients with prostate cancer experiencing acute urinary retention requiring catheterization successfully discontinued catheterization following bilateral orchiectomy [25]. Goserelin administration for 3 months has also been reported to significantly alleviate LUTS, and combining ADT with radiation therapy has demonstrated reductions in urinary retention incidence and LUTS severity [17]. Recent evidence further corroborates that ADT substantially alleviates LUTS in patients with prostate cancer, as reflected by reductions in total IPSS and its subscores, increased urinary flow rates, and enhanced QoL. These therapeutic benefits are most pronounced in patients presenting with moderate-to-severe baseline symptoms and typically manifest within 12 to 24 weeks following ADT initiation, underscoring its clinical utility in this specific patient population [19,26].

In this study, ADT induced significant increases in Qmax by 35.41% and 50.18% at 12 and 24 weeks, respectively, accompanied by concomitant and significant reductions in RU by 51.38% and 72.17% during the same periods. When stratified by baseline IPSS scores into mild and moderate-to-severe LUTS groups, both cohorts demonstrated significant reductions in PV and RU, alongside increases in Qmax, at both follow-up intervals. ADT also significantly improved LUTS, as evidenced by substantial mean reductions in total IPSS, voiding and storage subscores, and QoL scores compared to baseline at 12 and 24 weeks post-treatment (all *p* < 0.001). These findings suggest that ADT provides both short- and mid-term symptomatic relief and enhances QoL for patients with prostate cancer and LUTS. However, distinct patterns were observed when examining IPSS changes between the groups. Significant decreases in IPSS total score, IPSS voiding subscore, and IPSS storage subscore were observed at 12 and 24 weeks post-ADT in the moderate-to-severe LUTS group compared to baseline (*p* < 0.001). Conversely, in the mild LUTS group, IPSS total score, IPSS voiding subscore, and IPSS storage subscore increased at both 12 and 24 weeks post-ADT compared to baseline (*p* < 0.05).

This finding suggests that patients with mild baseline symptoms have limited potential for measurable improvement. IPSS reductions are typically more pronounced in individuals with higher baseline scores due to their greater symptom burden and broader range for discernible improvement post-intervention. Conversely, patients presenting with mild symptoms at baseline often approach the lower limit of the IPSS scale; thus, limiting the measurable extent of symptom improvement (i.e., a floor effect) [26,27]. Beyond this limited capacity for improvement, the observed increase in IPSS after ADT in the mild LUTS group could also be attributed to testosterone deprivation. Significant reductions in serum testosterone, a direct consequence of ADT, can negatively impact bladder function, potentially leading to elevated IPSS following treatment in this specific cohort. Although ADT effectively reduces prostate size and alleviates urinary symptoms, these hormonal alterations—particularly testosterone reduction—might paradoxically exacerbate LUTS in patients with mild symptoms, especially those with smaller prostates. Consequently, the overall IPSS in this specific subgroup may increase rather than decrease following treatment [26].

Higher baseline PSA levels typically indicate a larger PV and greater androgenic stimulation. As PSA is secreted by prostate epithelial cells, its concentration closely associates with the amount of androgen-dependent prostate tissue. Consequently, elevated pre-ADT PSA levels often suggest a larger PV and more pronounced urethral compression. Following ADT, prostate atrophy alleviates this urethral obstruction, leading to significant improvement in Qmax. Mechanistically, each 1 ng/mL increase in pre-ADT PSA associates with a greater degree of prostate tissue regression, which proportionally enhances Qmax [25,26]. Similarly, aging affects bladder outlet obstruction and its response to ADT. Although testosterone levels decline with age, PV generally increases, and the proportion of hormone-sensitive epithelial tissue becomes higher in older individuals. This combination often leads to more severe baseline bladder outlet obstruction in older individuals. ADT induces regression of the enlarged, androgen-dependent tissue; thereby, relieving urethral compression and improving Qmax. Age-related changes in the stromal-to-epithelial ratio may also increase sensitivity to hormone blockade, resulting in progressively greater Qmax improvement with advancing age [19,26].

In this study, each 1 ng/mL increase in pre-ADT PSA corresponded to a 0.023 mL/s increase in Qmax at 12 weeks post-ADT (β = 0.0231, *p* < 0.001) and a 0.015 mL/s increase at 24 weeks post-ADT (β = 0.0154, *p* < 0.001). Furthermore, each 1-year increase in baseline patient age was associated with a 0.23 mL/s increase in Qmax at 24 weeks post-ADT (β = 0.227, *p* = 0.043). These results indicate that both pre-ADT PSA and patient age can serve as valuable indirect predictors of the degree of urinary flow improvement after ADT.

ADT effectively alleviates LUTS in patients with prostate cancer by substantially reducing PV and improving urinary flow parameters. These therapeutic benefits are particularly pronounced in patients with larger baseline PVs and moderate-to-severe LUTS, reflected by significant improvements in Qmax, RU, and IPSS within 12 to 24 weeks after treatment. Conversely, patients with mild baseline LUTS may experience limited or no symptom improvement, or even worsening, due to the poor floor effect and potential hormonal influences. Furthermore, higher baseline PSA levels and older age emerged as independent predictors of greater Qmax improvement, underscoring their value in anticipating treatment responsiveness. Overall, ADT reduces tumor burden and provides meaningful symptomatic relief from urinary obstruction; thereby, enhancing the QoL in appropriately selected patients.

Despite the observed improvements in voiding symptoms and prostate volume reduction, it is crucial to acknowledge the potential adverse effects of ADT on the genitourinary system and overall quality of life. Testosterone suppression is well-documented to cause significant declines in libido and erectile function, which can adversely affect patients’ psychological well-being [16]. Furthermore, long-term androgen deprivation may induce urogenital atrophy, potentially impacting urethral compliance. Therefore, while ADT effectively alleviates LUTS in patients with prostate cancer, clinicians must carefully weigh these symptomatic benefits against the potential for sexual dysfunction and other systemic side effects when counseling patients.

This study has several limitations. First, the 24-week follow-up may be insufficient to capture long-term changes in urinary function or delayed adverse effects of ADT. While significant initial improvements were observed, the sustainability of these effects and potential late-onset compensatory changes in the bladder require further investigation through extended follow-up. Second, the single-center design with a modest sample size (n = 104) introduces the possibility of selection bias and may limit the generalizability of our findings to a broader, more diverse patient population. Specifically, we acknowledge the disparity in sample sizes between the mild (n = 18) and moderate-to-severe (n = 86) LUTS groups. However, this distribution was not arbitrary but reflects real-world clinical practice based on the IPSS diagnostic criteria, where patients requiring ADT typically present with more advanced age and symptoms. Furthermore, we confirmed that our results remained statistically robust across both parametric and non-parametric analyses, mitigating concerns regarding this imbalance. To address these limitations, a prospective multicenter cohort study with extended follow-up, predefined assessment schedules, and a prespecified intention-to-treat analysis with robust missing-data handling is planned to strengthen causal inference and long-term safety evaluation.

## 5. Conclusions

ADT significantly reduced PV and improved LUTS and urodynamic parameters in patients with prostate cancer. These therapeutic benefits were particularly evident in individuals presenting with moderate-to-severe baseline symptoms, resulting in enhanced urinary flow and QoL within a short- to mid-term period following treatment initiation.

## Figures and Tables

**Table 1:** Baseline demographics and clinical characteristics of the study population (N = 104).

Variables	Median [IQR]
Age (years), median (IQR)	76.00 (71.00–80.25)
BMI (kg/m^2^), median (IQR)	23.34 (21.78–26.08)
Gleason score (Pattern), n (%)	
6 (3 + 3)	14 (13.46%)
7 (3 + 4)	5 (4.81%)
7 (4 + 3)	10 (9.62%)
8 (4 + 4)	30 (28.85%)
9 (4 + 5)	14 (13.46%)
9 (5 + 4)	18 (17.31%)
10 (5 + 5)	13 (12.50%)
T stage, n (%)	
T2	27 (25.96%)
T3	45 (43.27%)
T4	32 (30.77%)
PV (cm^3^), median (IQR)	43.10 (32.89–61.95)
PSA (ng/mL), median (IQR)	42.25 (16.42–100.00)
Qmax (mL/s), median (IQR)	9.10 (5.10–15.25)
RU (mL), median (IQR)	50.00 (18.00–110.00)
IPSS total score, median (IQR)	23.00 (13.00–29.00)
IPSS voiding subscore, median (IQR)	14.00 (6.00–17.00)
IPSS storage subscore, median (IQR)	9.00 (7.50–15.00)
QoL score, median (IQR)	4.00 (4.00–5.00)

Note: Continuous variables are presented as median (interquartile range [IQR]), and categorical variables are expressed as number (percentage). Abbreviations: BMI, body mass index; IPSS, International Prostate Symptom Score; PSA, prostate-specific antigen; PV, prostate volume; Qmax, maximal uroflow rate; QoL, quality of life; RU, post-void residual urine volume.

**Table 2:** Comparison of changes following ADT between mild and moderate-to-severe LUTS groups (median [IQR]).

Variables	Mild LUTS Group (n = 18)	Moderate-to-Severe LUTS Group (n = 86)	*p*-Value
Changes from baseline to week 12			
Change in IPSS total score	3.50 (0.00–6.00)	−17.50 (−23.00–−11.00)	<0.001
Change in IPSS voiding subscore	1.00 (−0.75–5.00)	−11.00 (−14.00–−7.00)	<0.001
Change in IPSS storage subscore	1.00 (0.00–3.00)	−6.00 (−9.00–−3.00)	<0.001
Change in QoL score	−1.00 (−2.00–0.00)	−1.00 (−3.00–0.00)	<0.001
Changes from baseline to week 24			
Change in IPSS total score	3.00 (0.00–6.00)	−19.50 (−26.00–−12.25)	<0.001
Change in IPSS voiding subscore	0.00 (0.00–4.50)	−13.00 (−17.00–−8.00)	<0.001
Change in IPSS storage subscore	1.50 (0.25–4.00)	−6.50 (−9.00–−3.00)	<0.001
Change in QoL score	−2.00 (−3.00–0.00)	−3.50 (−4.00–−2.00)	<0.001

Note: Data are presented as median (interquartile range [IQR]). *p*-values were determined using the Mann–Whitney U test for comparing changes from baseline between the two groups. Abbreviations: ADT, androgen deprivation therapy; IPSS, International Prostate Symptom Score; LUTS, lower urinary tract symptoms; QoL, quality of life.

**Table 3:** Univariate and multivariate logistic regression analysis for predictors of significant LUTS improvement at 24 weeks.

Variable	Univariate OR (95% CI)	*p*-Value	Multivariate OR (95% CI)	*p*-Value
Age	1.001 (0.940–1.067)	0.965	1.029 (0.955–1.109)	0.452
BMI	1.034 (0.930–1.150)	0.538	1.037 (0.924–1.164)	0.536
Baseline PSA	1.000 (0.998–1.003)	0.929	1.000 (0.998–1.003)	0.801
Baseline PV	0.998 (0.985–1.012)	0.823	0.996 (0.982–1.011)	0.618
Gleason Score	0.985 (0.669–1.450)	0.938	0.967 (0.636–1.471)	0.876
T stage (T3 vs. T2)	2.118 (0.713–6.293)	0.177	2.302 (0.720–7.364)	0.16
T stage (T4 vs. T2)	2.753 (0.787–9.633)	0.113	3.407 (0.854–13.600)	0.083

Note: OR, odds ratio; CI, confidence interval. Abbreviations: BMI, body mass index; LUTS, lower urinary tract symptoms; PSA, prostate-specific antigen; PV, prostate volume.

## Data Availability

The data presented in this study are available on request from the corresponding author.
